# Special Issue “Research Advances on Cystic Fibrosis and CFTR Protein”

**DOI:** 10.3390/ijms26146708

**Published:** 2025-07-13

**Authors:** Debora Baroni

**Affiliations:** Istituto di Biofisica, Consiglio Nazionale delle Ricerche (CNR), Via De Marini, 6, 16149 Genova, Italy; debora.baroni@ibf.cnr.it; Tel.: +39-0106475559; Fax: +39-0106475500

## 1. Introduction

Cystic fibrosis (CF) is the most common life-limiting autosomal recessive disorder among Caucasian populations, with an incidence of 1 in 2500–3500 live births across Europe [1]. It is caused by mutations in the CFTR (Cystic Fibrosis Transmembrane Conductance Regulator) gene, which encodes a chloride and bicarbonate channel localised to the apical membrane of epithelial cells [2]. Deficient CFTR function results in dehydrated, viscous secretions, primarily affecting the respiratory tract. This leads to chronic pulmonary infection, inflammation, and, ultimately, respiratory failure [3]. Approximately 85% of patients also present with exocrine pancreatic insufficiency, contributing to malabsorption, nutritional deficiencies, and growth retardation [4]. Additional manifestations include hepatobiliary disease, CF-related diabetes, and male infertility due to the congenital bilateral absence of the vas deferens [5]. Patients typically exhibit elevated sweat chloride levels [6]. While the majority of cases present with a classical multisystem phenotype in infancy or early childhood, milder and atypical presentations are also recognised [7].

CF follows an autosomal recessive pattern of inheritance. Affected individuals inherit two defective CFTR alleles, one from each parent [8]. The estimated carrier frequency in Italy is approximately 1 in 25–30 individuals [9]. Carrier rates vary across Europe, ranging from approximately 1 in 22 to 1 in 25 in Northern Europe (e.g., the UK, Scandinavia, and Ireland), to 1 in 25 to 1 in 28 in Central Europe (Germany and the Benelux countries), and from 1 in 28 to 1 in 35 in Southern Europe (Spain, Italy, and Greece) [10]. Diagnoses are confirmed upon an observation of elevated sweat chloride levels and after genetic testing for pathogenic CFTR mutations [11]. Newborn screening has been widely implemented to facilitate the early detection of and interventions for this disorder [12].

The discovery of the CFTR gene in 1989 laid the foundation for a molecular understanding of CF and led to a search for targeted therapies [13]. In the following decades, incremental advancements in supportive care began to significantly prolong the associated life expectancy; however, the root cause of the disease still had not been determined. A transformative shift in CF management has been underway throughout the last decade, with the introduction of small-molecule drugs targeting the underlying molecular defects of CFTR [14,15]. These modulators include potentiators, which enhance channel gating, and correctors, which improve protein folding and trafficking [16,17,18]. This therapeutic revolution began with the development of high-throughput screening assays that enabled the identification of compounds capable of restoring CFTR activity [19]. Ivacaftor (VX-770), the first approved CFTR potentiator (Kalydeco), received FDA approval in 2012 for patients carrying the G551D gating mutation [20,21,22]. Subsequently, lumacaftor (VX-809) and tezacaftor (VX-661) were developed as correctors and combined with ivacaftor to treat patients who were homozygous for the F508del mutation [23,24,25,26]. However, the clinical benefits of these combinations were limited [27]. A major breakthrough occurred with the approval of the triple combination therapy elexacaftor/tezacaftor/ivacaftor (Trikafta/Kaftrio), which yielded substantial clinical improvements in patients with at least one F508del allele [28]. This regimen was approved by the FDA in 2019 and subsequently authorised by the EMA for adult and paediatric use [29].

CFTR modulator therapy now represents the standard of care for the majority of CF patients, covering 70–90% of genotypes depending on regional mutation distributions. Nonetheless, numerous CF-causing variants remain unresponsive to existing therapies, particularly minimal function mutations such as premature stop codons or splicing defects [30]. This underscores the critical need for ongoing mechanistic research to be conducted and for the development of next-generation therapeutics specifically tailored to rare and refractory mutations. Achieving progress in CF care depends not only on pharmacological innovation but also on the ability for robust basic and translational research to be carried out. In vitro models, including patient-derived airway cultures and intestinal organoids, have been pivotal in characterising CFTR dysfunction and evaluating pharmacological responses. Recent advancements in omics technologies, RNA-based therapeutics, and personalised medicine approaches have reshaped the landscape of CF research. In this context, “theratyping”, the functional classification of mutations based on their in vitro responsiveness to modulators, has emerged as a key strategy through which to increased access to precision therapies [31].

## 2. Scope and Significance of This Special Issue

This Special Issue of the *International Journal of Molecular Sciences* presents a collection of studies that strengthen our molecular understanding of CFTR biology and that contribute to the development of tailored therapies for cystic fibrosis. The articles included span a broad spectrum of experimental approaches and clinical insights, reflecting the expanding dimensions of CF research. Key topics include the pharmacodynamics of a novel CFTR activator, the elucidation of the mechanisms of action of the corrector elexacaftor (VX-445), CFTR expression in immune cells, virus–host interactions in the context of CFTR dysfunction, and in vitro theratyping using patient-derived models. Collectively, these contributions exemplify the Special Issue’s aim to stimulate discourse and innovation in the mechanistically driven and genotype-specific therapeutic strategies for CF. We hope that the insights provided will help to inform translational research, guide clinical applications, and ultimately contribute to improving outcomes for individuals with cystic fibrosis.

## 3. Overview of Contributions

### 3.1. Dissecting Acute CFTR Responses to VX-770, Cact-A1, and cAMP Elevation

In the article “VX-770, Cact-A1, and Increased Intracellular cAMP Have Distinct Acute Impacts upon CFTR Activity”, Nick and colleagues [32] investigate the differential acute effects of three pharmacological agents, the potentiator VX 770 (ivacaftor), the activator Cact-A1, and the forskolin/IBMX combination, on CFTR-mediated chloride transport. The present study employs both primary human nasal epithelial cells (HNECs) and Fischer rat thyroid (FRT) epithelial cells stably expressing wild-type CFTR, with the aim to assess CFTR activity using Ussing chamber electrophysiology. The researchers’ findings demonstrate that the magnitude and kinetics of CFTR activation are contingent on the specific compound applied, the sequence of exposure, and the physiological context, including ionic gradients and the intrinsic functional status of CFTR. It is noteworthy that elevating intracellular cAMP levels via forskolin/IBMX appears to induce additional cellular responses beyond CFTR activation, thereby altering the electrochemical driving force for chloride transport. Cact-A1, a novel activator with a cAMP-independent mechanism, exhibits distinct modulatory properties, highlighting the complex pharmacodynamics of CFTR under varying conditions.

Once the efficacy of the novel activator Cact-A1 has been demonstrated in epithelial models harbouring clinically relevant CFTR mutations, such as F508del or gating variants, its therapeutic potential, whether employed as a monotherapy or in combination with established potentiators, can be ascertained. Such validation would represent a significant step forward in expanding the pharmacological toolkit available for addressing CFTR dysfunction, particularly in individuals unresponsive to currently approved modulators.

### 3.2. CFTR Expression in Immune Cells: Rigorous Molecular Assessment Reveals Pitfalls and Challenges

Schnell and colleagues [33] conducted a comprehensive analysis of CFTR gene and protein expression in peripheral blood mononuclear cells (PBMCs), employing highly optimised protocols for quantitative PCR and Western blotting. The study detected low but measurable levels of CFTR mRNA in select immune cell populations, including CD4^+^ T cells, natural killer (NK) cells, and differentiated THP-1 and Jurkat cell lines. A key and novel finding was the detection of CFTR-like immune-reactive bands in the Western blots, which were subsequently identified as false positives through peptide competition and PNGase digestion. These artefacts, likely arising from proteins sharing structural epitopes or non-specific glycosylation-dependent interactions, underscore the interpretive challenges inherent to CFTR protein detection in non-epithelial systems.

The results highlighted in this study are of considerable significance to the broader cystic fibrosis research community. The authors convincingly underscore the necessity of orthogonal validation strategies, such as the inclusion of negative controls, epitope masking, and enzymatic digestion, when assessing CFTR expression, particularly in non-epithelial or low-abundance cellular contexts. This rigorous methodological framework provides a solid foundation for future investigations aimed at clarifying the potential functional roles of CFTR in immune cell subsets under pathological conditions, such as infection or inflammation. Moreover, it paves the way for the transcriptomic and proteomic profiling of CFTR-related signatures in peripheral immune cells of CF patients, with the potential to aid in the identification of novel biomarkers of disease activity or therapeutic response.

### 3.3. The Activation of Cellular Senescence Pathways in CFTR-Defective Cells Is Reversed by SARS-CoV-2 Infection

Merigo and colleagues [34] reveal novel findings regarding the interaction between CFTR deficiency and host–virus dynamics, examining the cellular mechanisms underlying the differential impact of SARS-CoV-2 infection on wild-type and CFTR knockout (KO) 16HBE14o^−^ bronchial epithelial cells, with a specific focus on senescence-related pathways. Their study demonstrates that CFTR-deficient cells exhibit elevated markers of senescence (p21 expression) and reduced proliferative activity (Ki67 expression) under basal conditions. Intriguingly, upon SARS-CoV-2 infection, these senescence features were reversed in CFTR KO cells, with p21 levels decreasing, Ki67 expression increasing, and viral gene expression significantly attenuated compared to that of wild-type cells. Transmission electron microscopy further revealed lipid accumulation in CFTR-deficient cells, including lipolysosomes and residual bodies.

These findings suggest that CFTR dysfunction modulates the host cell’s antiviral response and may help explain the unexpectedly milder clinical course of COVID-19 observed in many CF patients. While these in vitro observations are compelling, future studies should assess their relevance in primary human airway epithelia and patient-derived models. In addition, elucidating the broader role of CFTR in regulating senescence and antiviral signalling may uncover novel therapeutic targets for respiratory infections in CF and beyond.

### 3.4. Functional and Clinical Evaluation of CFTR Modulators in Organoids and Rectal Biopsies from a Patient with N1303K/Class I CFTR Variants

In their study, Efremova and colleagues [35] evaluate the therapeutic potential of elexacaftor/tezacaftor/ivacaftor (ETI) in an individual harbouring the challenging N1303K/class I CFTR genotype. The N1303K variant, located within nucleotide-binding domain 2 (NBD2), is historically associated with limited responsiveness to modulators. Integrating data from patient-derived intestinal organoids, intestinal current measurements (ICMs) from rectal biopsies, and longitudinal clinical follow-up, the authors provide compelling evidence of partial CFTR function restoration following ETI treatment. Organoid swelling assays revealed a significant increase in CFTR-dependent fluid secretion upon ETI exposure; in contrast, dual combinations such as VX-770/VX-809 or VX-770/VX-661 have proven inefficacious. These findings were corroborated by enhanced CFTR-mediated ion transport in ICM analysis and modest, yet clinically meaningful, improvements in pulmonary function, nutritional status, and respiratory symptoms.

Interestingly, sweat chloride concentrations did not improve, emphasising tissue-specific differences in CFTR modulation and the need to reassess classical biomarkers of treatment efficacy. This study paves the way for further investigations into whether prolonged treatment, optimised dosing strategies, or adjunctive therapies could enhance the clinical impact of ETI in patients with N1303K or similarly refractory CFTR genotypes. These efforts are critical for advancing the goals of precision medicine and expanding access to effective therapies for all individuals with CF.

### 3.5. Mechanistic Insights into VX445 Binding to F508del CFTR

In their mechanistic investigation, Bongiorno and colleagues [36] provide valuable insights into the molecular mode of action of elexacaftor (VX-445), a core component of the triple combination modulator therapy Kaftrio/Trikafta. The authors investigated the domain-specific activity of VX-445 in depth to clarify its contribution to the rescue of F508del-CFTR, the most prevalent CF-causing mutation. Using molecular and biochemical assays in heterologous systems, they showed that VX-445 selectively enhances the expression and maturation of membrane-spanning domain 2 (MSD2), thereby identifying this region as a key pharmacological target.

Importantly, VX-445 displayed additive effects on F508del-CFTR rescue when combined with either type I or type II correctors, supporting the hypothesis that it operates through a distinct and complementary mechanism. These findings validate the rationale for combinatorial regimens and form a framework for the development of next-generation domain-specific correctors. The identification of MSD2 as a pharmacologically responsive region opens avenues for rational structure-based drug design, particularly for rare CFTR variants with domain-localised misfolding defects.

## 4. Future Perspectives and Concluding Remarks

Over the past two decades, substantial improvements in cystic fibrosis (CF) care have been achieved through an integrated therapeutic approach combining CFTR modulators with optimised anti-infective regimens, anti-inflammatory therapies, nutritional support, and physiotherapy [37]. These advances have dramatically altered the clinical trajectory of CF, transforming it from a fatal paediatric disease into a chronic condition with markedly improved prognosis and quality of life.

Nevertheless, the path forward presents several critical challenges. Although modulator therapy now benefits the majority of patients, individuals harbouring rare, ultra-rare, or minimal function mutations remain without effective options [38]. Future efforts should therefore focus on the development of next-generation correctors and potentiators, as well as alternative strategies such as RNA-based therapies, readthrough agents, and personalised theratyping platforms based on patient-derived models or gene editing technologies.

At the same time, there is an increasing need to reassess and refine the biomarkers used to monitor therapeutic efficacy. As some studies in this Special Issue highlight, traditional readouts, such as sweat chloride concentration, may not adequately reflect CFTR activity across tissues or in response to mutation-specific treatments. Multi-parametric assessment tools, including transcriptomic and proteomic signatures, hold promise for improving patient stratification and guiding treatment decisions [39].

Finally, the widespread adoption of modulator therapy poses significant challenges in terms of costs and global access [40]. The annual price of elexacaftor/tezacaftor/ivacaftor therapy exceeds USD 300,000 in the United States and ranges between EUR 150,000 and 200,000 in Europe. These figures raise fundamental questions about the long-term sustainability of care and equitable access across healthcare systems [41]. Strategies to promote affordability, including the development of generic alternatives, public–private partnerships, and equitable reimbursement models, will be essential to ensuring that the benefits of scientific progress are shared globally.

In conclusion, the contributions featured in this Special Issue highlight the multidisciplinary and translational nature of modern CF research. They illustrate how insights from molecular biology, pharmacology, immunology, and clinical practice can converge to inform more effective, personalised, and accessible therapeutic paradigms. Continued investment in fundamental research, innovation in disease modelling, and commitment to healthcare equity will be pivotal in shaping the future of CF care.
